# Control Power in Continuous Variable Controlled Quantum Teleportation

**DOI:** 10.3390/e26121017

**Published:** 2024-11-25

**Authors:** Yuehan Tian, Dunbo Cai, Nengfei Gong, Yining Li, Ling Qian, Runqing Zhang, Zhiguo Huang, Tiejun Wang

**Affiliations:** 1 State Key Laboratory of Information Photonics and Optical Communications, Beijing University of Posts and Telecommunications, Beijing 100876, China; 2 School of Science, Beijing University of Posts and Telecommunications, Beijing 100876, China; 3 China Mobile (Suzhou) Software Technology Company Limited, Suzhou 215163, China

**Keywords:** continuous variable quantum systems, controlled quantum teleportation, control power

## Abstract

Controlled quantum teleportation is an important extension of multipartite quantum teleportation, which plays an indispensable role in building quantum networks. Compared with discrete variable counterparts, continuous variable controlled quantum teleportation can generate entanglement deterministically and exhibit higher superiority of the supervisor’s authority. Here, we define a measure to quantify the control power in continuous variable controlled quantum teleportation via Greenberger–Horne–Zeilinger-type entangled coherent state channels. Our results show that control power in continuous variable controlled quantum teleportation increases with the mean photon number of coherent states. Its upper bound is 1/2, which exceeds the upper bound in discrete variable controlled quantum teleportation (1/3). The robustness of the protocol is analyzed with photon absorption. The results show that the improving ability of the control power will descend by the increasing photon loss, with the upper bound unchanged and robust. Our results illuminate the role of control power in multipartite continuous variable quantum information processing and provide a criterion for evaluating the quality of quantum communication networks.

## 1. Introduction

Quantum teleportation, as a crucial role of quantum communication, can transfer the quantum states of the particle instead of a real particle [1,2,3]. Therefore, quantum teleportation is able to connect the units of quantum information processing, contributing to building quantum networks [4,5]. The proposal for quantum teleportation is initially reported in discrete variable (DV) quantum system [1], and then expended in a continuous variable (CV) quantum system [6,7,8,9,10]. In DV quantum information processing systems, maximum entanglement can be achieved, but the generation of entanglement is usually probabilistic. By contrast, in CV systems, entanglement can be generated deterministically at the cost of losing perfect entanglement [7,9,11]. In addition, high-efficiency homodyne detections of CV systems can realize the tests free of postselection [12]. Nowadays, combining these advantages and disadvantages, quantum information processing including teleportation in both of DV and CV systems is developing simultaneously [13,14,15,16,17].

In the realm of quantum networks, supervision, control, and collaborative efforts among various parties constitute foundational pillars. These encompass vigilant monitoring of the operational status of quantum servers, anticipating and responding to potential crises, and orchestrating network adjustments to optimize the utilization and efficiency of its resources [16]. Quantum networks with three parties are explored for communication schemes, such as quantum secret sharing [18,19] and controlled quantum teleportation (CQT) [20]. In these applications, the security of communication is guaranteed by a third party. A supervisor, which is the third party, has the ability to either authorize or prohibit the successful transmission of a quantum state from the sender to the receiver, but is not the owner of the information transferred. Controlled quantum teleportation represents a sophisticated variation and extension of the conventional quantum teleportation protocol [21,22], requiring the explicit authorization and involvement of a supervisor for its execution. The existence of eavesdroppers or untrusted receivers can be certified by the supervisor in the device-independent way [23,24] because of the genuine multipartite quantum nonlocality [25,26] in CQT channels. Thus far, experimental realizations of both the CV and DV CQT protocol have been reported in optical systems [3,27,28]. As an important indicator for evaluating the performance of quantum networks, control power is discussed in detail for DV quantum information processing [23,29,30,31]. Nevertheless, in the context of CV quantum networks, the control power also emerges as a significant and noteworthy criterion for evaluation, which has not discussed up to now.

Entangled coherent states serve as important quantum resources, and have been instrumental in numerous quantum protocols, including quantum teleportation [32,33,34,35], controlled quantum teleportation [36,37,38], and quantum key distribution [39,40]. In this paper, we define a measure to quantify the control power in CV CQT via three-mode maximally entangled Greenberger–Horne–Zeilinger-type (GHZ-type) entangled coherent state channels. The influence of information parameter θ and mean photon number of coherent states ${\left|\alpha \right|}^{2}$ is studied with the fidelity and control power. When the mean photon number is small (${\left|\alpha \right|}^{2}<2$), the fidelities change with information parameter θ. When ${\left|\alpha \right|}^{2}\geq 2$, the influence of information parameter θ almost disappears. Our results show that control power in CV CQT increases with the mean photon number of coherent states and then approaches an upper bound 1/2. In addition, the robustness of the protocol is analyzed with photon absorption. It is found that the increasing photon loss will reduce the improving ability of the control power without changing its upper bound. When the survived photon proportion η=1.0, the control power reaches the upper bound with ${\left|\alpha \right|}^{2}\approx 6$. When the photon loss increases (for example η=0.7), the control power obtains the upper bound with a larger mean photon number (${\left|\alpha \right|}^{2}\approx 8$).

The present paper is structured as follows. In Section 2, we review a CV CQT protocol for Alice to teleport a qubit encoded by superposition of phase opposite coherent states to Bob with the assistance of Charlie when the three parties share a prior three-mode GHZ-type entangled coherent state. In Section 3, control power of the supervisor in CV controlled teleportation is calculated. Section 4 analyzes the robustness with photon absorption. Finally, the summary and outlook are drawn in Section 5.

## 2. Continuous Variable Quantum Controlled Teleportation via GHZ-Type Entangled Coherent State Channels

**Figure 1:**
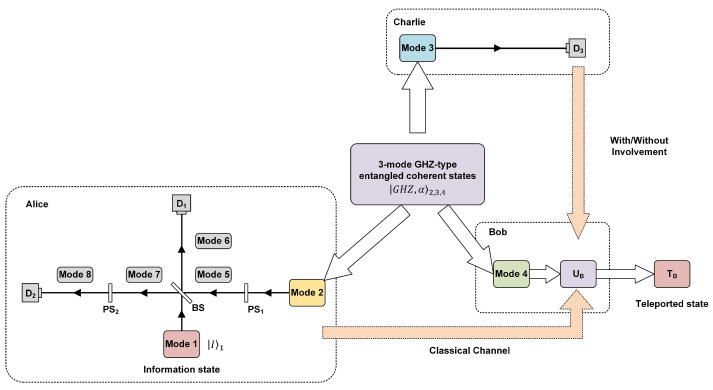
Schematic of the proposed scheme for the CQT of superposed coherent states using three mode GHZ-type entangled coherent state. The sender (Alice) mixes the information state, ${|I\rangle }_{1}$ in mode 1 with mode 2 of GHZ-type entangled coherent state ${|GHZ,\alpha \rangle }_{2,3,4}$ using symmetric beam splitter (BS) and two phase shifters ($PS_{1}$ and $PS_{2}$). Then, Alice performs photon counting (PC) measurements on the output modes 6 and 8 using detectors $D_{1}$ and $D_{2}$, respectively. In addition, Alice communicates her PC outcome to the receiver (Bob) using classical channel. The supervisor (Charlie) executes a PC measurement on mode 3 using detector $D_{3}$, and communicate the outcome to Bob using classical channels. According to the received classical inputs, Bob implements appropriate unitary operation $U_{B}$ on modes 4, which finally completes the teleportation protocol with the teleported state $T_{B}$.

In this section, a continuous variable quantum controlled teleportation protocol via GHZ-type entangled coherent state channels is reviewed, which is proposed by Pandey et al. [41], as given in Figure 1. The protocol is selected because it is a simple enough quantum teleportation network model to benefit the analyze of control power of CV controlled teleportation. Contributing to improve the security of the protocol, there is a supervisor Charlie in the system.

A sender Alice has a single qubit information to be teleported in mode 1, which is encoded in superposition of phase opposite coherent states as

(1) $${|I\rangle }_{1}=\varepsilon_{+}{|\alpha \rangle }_{1}+\varepsilon_{-}{|-\alpha \rangle }_{1},$$

where αk=exp−α2α222∑n=0∞αnn!n is a coherent state in the Fock basis of amplitude α at the mode *k* in this paper. The normalization of the information state demands ε+2+ε−2+2x2Reε+∗ε−=1. Throughout the context, $x=e^{-{\left|\alpha \right|}^{2}}$. The information state can also be expressed by Bloch space representation in terms of even coherent states $|E\rangle$ and odd coherent states $|O\rangle$ [42] as follows:

(2) $${|I\rangle }_{1}=A_{+}{|E\rangle }_{1}+A_{-}{|O\rangle }_{1},$$

where

(3) $$\begin{array}{c} |E\rangle ={\left[\sqrt{2\left(1+x^{2}\right)}\right]}^{-1}\left(|\alpha \rangle +|-\alpha \rangle \right),\\ |O\rangle ={\left[\sqrt{2\left(1-x^{2}\right)}\right]}^{-1}\left(|\alpha \rangle -|-\alpha \rangle \right). \end{array}$$

$|E\rangle$ and $|O\rangle$ are well-known Schrödinger cat states [42]. θ and φ are the polar angle (θ∈[0,π]) and the azimuthal angle (φ∈[0,2π]) in Bloch space. Without loss in generality, the relationship of $A_{+}$, $A_{-}$ and θ, φ can be expressed by A+=cosθθ22,A−=eiφsinθθ22, satisfying the normalization condition ${\left|A_{+}\right|}^{2}+{\left|A_{-}\right|}^{2}=1$. The mutual conversion of non-orthogonal representation as Equation (1) and orthogonal representation as Equation (2) can realize flexibly using the interrelationship A±=ε+±ε−1±x21±x222 and $\varepsilon_{\pm }={\left[2\left(1+x^{2}\right)\right]}^{-1/2}A_{+}\pm {\left[2\left(1-x^{2}\right)\right]}^{-1/2}A_{-}$.

A three-mode maximally entangled GHZ-type entangled coherent states channel is shared among the three partners of the network, including the sender Alice, the receiver Bob, and the supervisor Charlie. The form of the GHZ-type channel is as follows:

(4) $$\begin{array}{c} {|GHZ,\alpha \rangle }_{2,3,4}={\left[2\left(1-x^{6}\right)\right]}^{-1/2}\cdot \left({|\alpha ,\alpha ,\alpha \rangle }_{2,3,4}-{|-\alpha ,-\alpha ,-\alpha \rangle }_{2,3,4}\right). \end{array}$$

The three partners share the modes of the channel, respectively, where Alice has mode 2, Bob has mode 3, and Charlie has mode 4.

In order to transmit the information state in mode 1 to the receiver Bob under the supervision of Charlie, the sender Alice mixes modes 1 and 2 using a symmetric beam splitter (BS), as shown in Figure 1. The BS is fitted with two phase shifters (PSs) at its second input ($PS_{1}$ at mode 2) and second output port ($PS_{2}$ at mode 7). The $PS_{1}$ makes state ${|\beta \rangle }_{2}$ become ${|-i\beta \rangle }_{5}$. The BS makes states ${|\delta ,\gamma \rangle }_{1,5}$ become ${|\frac{\delta +i\gamma }{\sqrt{2}},\frac{\gamma +i\delta }{\sqrt{2}}\rangle }_{6,7}$. The $PS_{2}$ makes state of mode 7, ${|\phi \rangle }_{7}$, becomes ${|-i\phi \rangle }_{8}$. Therefore, the scheme of one beam splitter and two phase shifters transforms the states in the inputs modes ${|\alpha ,\beta \rangle }_{1,2}$ to those in the output modes ${|\frac{\alpha +\beta }{\sqrt{2}},\frac{\alpha -\beta }{\sqrt{2}}\rangle }_{6,8}$. Alice performs a photon counting (PC) measurement on modes 6 and 8. The composite state evolution of system can be expressed as

(5) $$\begin{array}{c} \begin{array}{c} \;\;\;\;{|\psi \rangle }_{1,2,3,4}={|I\rangle }_{1}⊗{|GHZ,\alpha \rangle }_{2,3,4}\\ \;\;\;\;\;\;\;\;\;\;\;={\left[2\left(1-x^{6}\right)\right]}^{-1/2}[\varepsilon_{+}\left({|\alpha ,\alpha ,\alpha ,\alpha \rangle }_{1,2,3,4}-{|\alpha ,-\alpha ,-\alpha ,-\alpha \rangle }_{1,2,3,4}\right)\\ \;\;\;\;\;\;\;\;\;\;\;\;+\varepsilon_{-}\left({|-\alpha ,\alpha ,\alpha ,\alpha \rangle }_{1,2,3,4}-{|-\alpha ,-\alpha ,-\alpha ,-\alpha \rangle }_{1,2,3,4}\right)],\\ \underset{\to }{{PS}_{1}}\;\;\;{|\psi \rangle }_{1,5,3,4}={\left[2\left(1-x^{6}\right)\right]}^{-1/2}[\varepsilon_{+}\left({|\alpha ,-i\alpha ,\alpha ,\alpha \rangle }_{1,5,3,4}-{|\alpha ,i\alpha ,-\alpha ,-\alpha \rangle }_{1,5,3,4}\right)\\ \;\;\;\;\;\;\;\;\;\;\;+\varepsilon_{-}\left({|-\alpha ,-i\alpha ,\alpha ,\alpha \rangle }_{1,5,3,4}-{|-\alpha ,i\alpha ,-\alpha ,-\alpha \rangle }_{1,5,3,4}\right)],\\ \underset{\to }{BS}\;\;\;\;\;{|\psi \rangle }_{6,7,3,4}={\left[2\left(1-x^{6}\right)\right]}^{-1/2}[\varepsilon_{+}\left({|\sqrt{2}\alpha ,0,\alpha ,\alpha \rangle }_{6,7,3,4}-{|0,\sqrt{2}i\alpha ,-\alpha ,-\alpha \rangle }_{6,7,3,4}\right)\\ \;\;\;\;\;\;\;\;\;\;\;+\varepsilon_{-}\left({|0,-\sqrt{2}i\alpha ,\alpha ,\alpha \rangle }_{6,7,3,4}-{|-\sqrt{2}\alpha ,0,-\alpha ,-\alpha \rangle }_{6,7,3,4}\right)],\\ \underset{\to }{{PS}_{2}}\;\;\;{|\psi \rangle }_{6,8,3,4}={\left[2\left(1-x^{6}\right)\right]}^{-1/2}[\varepsilon_{+}\left({|\sqrt{2}\alpha ,0,\alpha ,\alpha \rangle }_{6,8,3,4}-{|0,\sqrt{2}\alpha ,-\alpha ,-\alpha \rangle }_{6,8,3,4}\right)\\ \;\;\;\;\;\;\;\;\;\;\;+\varepsilon_{-}\left({|0,-\sqrt{2}\alpha ,\alpha ,\alpha \rangle }_{6,8,3,4}-{|-\sqrt{2}\alpha ,0,-\alpha ,-\alpha \rangle }_{6,8,3,4}\right)]. \end{array} \end{array}$$

According to Prakash et al. [35], the photon counter is supposedly so sensitive that it can distinguish among a vacuum, non-zero even (NZE), and odd photons. The relationship is written as follows:

(6) $$\begin{array}{c} |\pm \sqrt{2}\alpha \rangle =x|0\rangle +\frac{1-x^{2}}{\sqrt{2}}|\sqrt{2}NZE\rangle \pm \sqrt{\frac{1-x^{4}}{2}}|\sqrt{2}O\rangle , \end{array}$$

where

(7) $$\begin{array}{c} |\sqrt{2}NZE\rangle =\frac{|\sqrt{2}\alpha \rangle +|-\sqrt{2}\alpha \rangle -2x|0\rangle }{\sqrt{2}\left(1-x^{2}\right)} \end{array}$$

is the normalized form of NZE number of photons. Subsequently, Alice communicates her PC results to Bob through a two-bit classical channel. Nevertheless, after receiving the PC result from Alice, Bob still needs the result from Charlie to perform a corresponding unitary operation to construct the original information state. Consequently, Charlie plays an indispensable role in the protocol by performing the photon counting on mode 3 to confirm an even or odd photon. Combining Equation (6), modes 6 and 8 are expanded in orthogonal basis $\left\{|0\rangle ,|\sqrt{2}NZE\rangle ,|\sqrt{2}O\rangle \right\}$. Meanwhile, modes 3 and 4 are expanded in the basis $\left\{|E\rangle ,|O\rangle \right\}$ using Equation (3). Therefore, the composite state of the system can be calculated as

(8) ψ6,8,3,4=  [2(1−x6)]−1/2·{x21−x2·[0608E3(A+O4)+0608O3(A+E4)]+1+x2·(1−x2)1+x2·(1−x2)22·[2NZE608E3·(A−1+x21−x2E4+A+1−x21+x2O4)+062NZE8E3·(−A−1+x21−x2E4+A+1−x21+x2O4)]+1−x2·(1−x2)1−x2·(1−x2)22·[2NZE608O3·(A+E4+A−O4)+062NZE8O3·(A+E4−A−O4)]+1−x2·(1+x2)1−x2·(1+x2)22·[2O608E3·(A+E4+A−O4)+062O8E3·(−A+E4+A−O4)]+1+x2·1−x21+x2·1−x222·[2O608O3·(A−1+x21−x2E4+A+1−x21+x2O4)+062O8O3·(A−1+x21−x2E4−A+1−x21+x2O4)]}.

The fidelity of this protocol is explored [41], evaluating the quality of the quantum teleportation preliminarily. However, in Ref. [41], the control power of the third party has not been discussed.

To elaborate on the controlling capability of the supervisor Charlie to this protocol, it is necessary to consider the probability of situations both with and without involvement of Charlie. It is assumed that the failed cases arise when the results of photon counting are vacuum states, otherwise they are successful cases. In order to separate the probability of failed case of Charlie, the state ${|E\rangle }_{3}$ can be divided into ${|0\rangle }_{3}$ and ${|NZE\rangle }_{3}$ for consideration. Therefore, Equation (3) can be rewritten as

(9) $$|E\rangle =\frac{\sqrt{2x}}{\sqrt{1+x^{2}}}|0\rangle +\frac{1-x}{\sqrt{1+x^{2}}}|NZE\rangle .$$

Correspondingly, the relationship among a vacuum, NZE and odd photons can be given as

(10) $$\begin{array}{c} |\pm \alpha \rangle =\sqrt{x}|0\rangle +\frac{\sqrt{2}}{2}\left(1-x\right)|NZE\rangle \pm \sqrt{\frac{1-x^{2}}{2}}|O\rangle . \end{array}$$

The unitary operations by Bob after receiving results of Charlie are still considered based on $|E\rangle$ and $|O\rangle$.

One can see from Equation (8) that, out of these two counts of modes 6 and 8, one count is always zero and the other can give a zero, NZE, or odd counts. Then, the collective PC result in modes (3, 6, 8) can be one of (0/NZE/ODD, 0, 0), (0/NZE/ODD, NZE, 0), (0/NZE/ODD, 0, NZE), (0/NZE/ODD, ODD, 0), and (0/NZE/ODD, 0, ODD). The PC measurement amounting to a total of 15 mutually exclusive outcomes, which we shall abbreviate by cases m=1,2,...,15 in Table A1 in Appendix A. It should be useful for the following discussions that the states with Bob $|B_{m}\rangle$ for case *m* can be written as

(11) $$\begin{array}{c} |B_{1}\rangle ={|E\rangle }_{4},\;|B_{2}\rangle ={|O\rangle }_{4},\\ |B_{3/5}\rangle =N_{4}\left[A_{+}\left(1-x^{2}\right){|O\rangle }_{4}\pm A_{-}\left(1+x^{2}\right){|E\rangle }_{4}\right],\\ |B_{4/6}\rangle =A_{+}{|E\rangle }_{4}\pm A_{-}{|O\rangle }_{4},\\ |B_{7/9}\rangle =A_{-}{|O\rangle }_{4}\pm A_{+}{|E\rangle }_{4},\\ |B_{8/10}\rangle =N_{4}\left[A_{-}\left(1+x^{2}\right){|E\rangle }_{4}\pm A_{+}\left(1-x^{2}\right){|O\rangle }_{4}\right], \end{array}$$

where $N_{4}={\left(1+x^{4}-2x^{2}\cos\theta \right)}^{-1/2}$ denotes the normalization parameter of mode 4 with Bob.

For information state $|\psi_{I}\rangle$ and teleported state $|\psi_{T}\rangle$, the fidelity of teleportation can be expressed by

(12) F=ψIψIψTψT2.

With the complete permission of the supervisor Charlie, the fidelity of the controlled teleportation will be unity or one of the following forms:

(13) F0=A−2=sin2θθ22,F1=1−x4sin2θx4sin2θ1+x4−2x2cosθ1+x4−2x2cosθ.

In the 15 cases from Table A1, it is noted that the teleported states for cases 1, 2, and 3 cannot be constructed same as the information states using any unitary transformation. These cases can be defined as wrong teleportation. When performing the corresponding operation, the remaining 12 cases amount to teleportation with fidelity either equal to unity or close to unity depending on mean photon number ${\left|\alpha \right|}^{2}$. These 12 cases will lead to correct controlled teleportation. The probability of cases 1, 2, and 3 is calculated as follows:

(14) P01=2x2x1+x21+x2·x2cos2θθ22x2cos2θθ221+x2+x41+x2+x4,P02=1−x21−x21+x21+x2·x2cos2θθ22x2cos2θθ221+x2+x41+x2+x4,P03=x2cos2θθ22x2cos2θθ221+x2+x41+x2+x4.

The probability of the cases when Alice receives either (NZE, 0) or (0, NZE) is one of the following expressions:

(15) P11=xx1+x21+x21+x4−2x2cosθ·1141+x2+x441+x2+x4,P12=1−x21−x21+x21+x21+x4−2x2cosθ·1181+x2+x481+x2+x4,P13=1−x221−x2281+x2+x481+x2+x4.

Additionally, the probability of the cases when Alice receives either (ODD, 0) or (0, ODD) is one of the following expressions:

(16) P21=x·1+x2x·1+x241+x2+x441+x2+x4,P22=1−x2·1+x2·1+x281+x2+x481+x2+x4,P23=1+x4−2x2cosθ1+x4−2x2cosθ81+x2+x481+x2+x4.

## 3. Control Power of the Supervisor in Continuous Variable Controlled Quantum Teleportation

**Figure 2:**
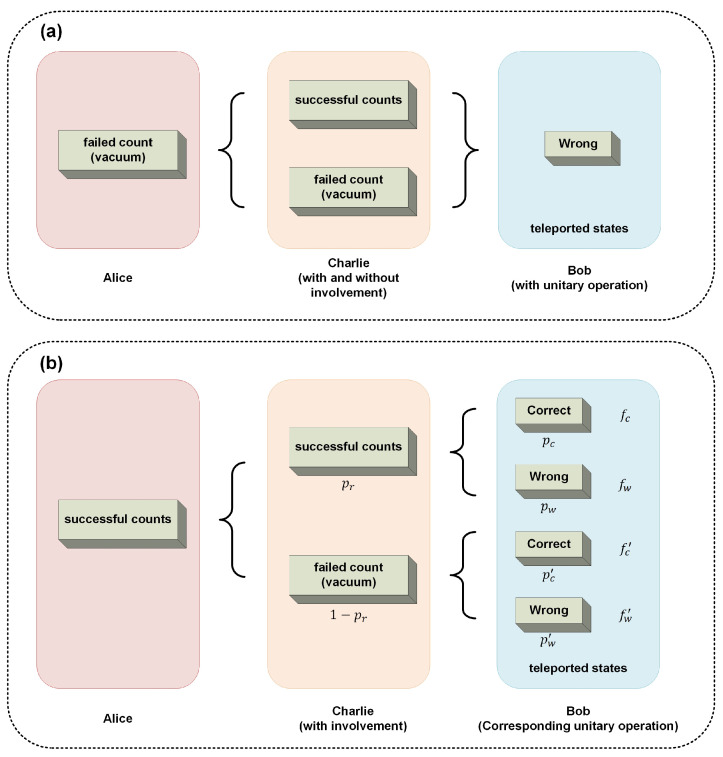
(**a**) The situations when Alice has failed counts. The teleportation will definitely be wrong regardless of results of Charlie. (**b**) The situations when Alice has successful counts with the involvement of the supervisor Charlie. The probability of the teleported states for Bob and the corresponding fidelity are indicated.

**Figure 3:**
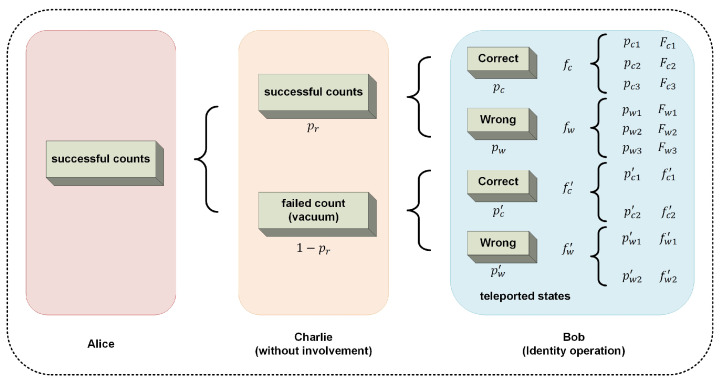
The situations when Alice has successful counts without the involvement of the supervisor Charlie. The probability of the teleported states for Bob and the corresponding fidelity are indicated.

In quantum networks, control power [22] can show the authority of the supervisor (Charlie) over the transmission and, hence, it is an important measure to validate the effectiveness of the scheme. In this section, the control power of the supervisor in continuous variable CQT is analyzed. Analogous with control power in discrete variable [23], the control power CP is defined as the difference between two different fidelities,

(17) CP=FCQT−FNC=∫02πdφ∫0πfCQT−fNCdθ∫02πdφ∫0πfCQT−fNCdθ∫02πdφ∫0πdθ∫02πdφ∫0πdθ=∫0πfCQT−fNCdθ∫0πfCQT−fNCdθππ=∫0πCP0dθ∫0πCP0dθππ,

where $F_{CQT}$ is the conditioned fidelity with the supervisor’s involvement, considering the cases that cause correct controlled teleportation. $F_{NC}$ is the non-conditioned fidelity, which is the fidelity without the supervisor’s involvement. ${CP}_{0}=f_{CQT}-f_{NC}$. To elaborate the algorithm of the fidelities $f_{CQT}$ and $f_{NC}$, related to information parameter θ, the situations whether Alice and Charlie have successful or failed count are described in Figure 2 and Figure 3. The corresponding notes of probability and fidelity are indicated in both figures. Notably, in the scheme, when the both PC results of Alice are vacuum as shown in Figure 2a, the average fidelity is given by

(18) F0¯=∫02πdφ∫0πF0dθ∫02πdφ∫0πF0dθ∫02πdφ∫0πdθ∫02πdφ∫0πdθ=1/2.

It is clear that the teleportation will definitely be wrong regardless of the results of Charlie and whether the control power of the supervisor is zero. Therefore, in the following discussion of control power, the cases that Alice has successful counts in Table A1 are considered, which means the PC counts of Alice are not (0, 0).

The situation of conditioned fidelity $F_{CQT}$ is discussed. As shown in Figure 2b, the expression of $f_{CQT}$ can be expanded as the average fidelity when Bob performs the corresponding unitary operations in Table A1 for all cases from 4 to 15, which can be written as

(19) $$\begin{array}{c} f_{CQT}=p_{r}\left(p_{c}f_{c}+p_{w}f_{w}\right)+\left(1-p_{r}\right)\left(p_{c}^{\prime }f_{c}^{\prime }+p_{w}^{\prime }f_{w}^{\prime }\right), \end{array}$$

where $p_{r}=1-x$ is the probability when Charlie records successful counts on the basis of Equation (10). $p_{c}$ and $p_{w}$ represent the probabilities of correct and wrong teleportation when Charlie receives successful counts, while $p_{c}^{\prime }$ and $p_{w}^{\prime }$ denote the probabilities of correct and wrong teleportation when the counting of Charlie fails. $f_{c}$ and $f_{w}$ are fidelities corresponding to the cases with $p_{c}$ and $p_{w}$, respectively. Likewise, $f_{c}^{\prime }$ and $f_{w}^{\prime }$ can be given according to the cases of correct and wrong teleportation with $p_{c}^{\prime }$ and $p_{w}^{\prime }$. The probabilities parameters are as follows:

(20) pc=∑m=6,9,11,14p(m)=2P13+2P22=1−x−x3+x41−x−x3+x421+x2+x421+x2+x4,pw=∑m=5,8,12,15p(m)=2P12+2P23=1+x4−2x2cosθ1−x21−x21+x21+x2+11+x4−2x2cosθ1−x21−x21+x21+x2+141+x2+x441+x2+x4,pc′=∑m=10,13p(m)=2P21=x1+x2x1+x221+x2+x421+x2+x4,pw′=∑m=4,7p(m)=2P11=1+x4−2x2cosθxx1+x21+x21+x4−2x2cosθxx1+x21+x241+x2+x441+x2+x4,

where $p_{\left(m\right)}$ denotes the probability of case *m*. From Table A1, one can clearly see that the fidelity of cases m=6,9,10,11,13,14 is unity, while the fidelity of cases m=4,5,7,8,12,15 is $F_{1}$. Then, the according fidelities are $f_{c}=f_{c}^{\prime }=1$ and $f_{w}=f_{w}^{\prime }=F_{1}$.

The situation of non-conditioned fidelity $F_{NC}$ is elaborated. Without the involvement of supervisor, the non-conditioned fidelity can be regarded as equivalent with the weighted average fidelity when Bob performs the same unitary operations for all cases from 4 to 15. Without loss of generality, we select identity transformation *I* as the unitary operation of Bob. Thus, the forms of teleported states are the same with Bob’s states $|B_{m}\rangle$ in Table A1. The expression of $f_{NC}$ in the protocol is given by

(21) $$\begin{array}{c} f_{NC}=p_{r}\left[p_{c}\left(p_{c1}f_{c1}+p_{c2}f_{c2}+p_{c3}f_{c3}\right)+p_{w}\left(p_{w1}f_{w1}+p_{w2}f_{w2}+p_{w3}f_{w3}\right)\right]\\ \;\;\;\;\;\;\;+\left(1-p_{r}\right)\left[p_{c}^{\prime }\left(p_{c1}^{\prime }f_{c1}^{\prime }+p_{c2}^{\prime }f_{c2}^{\prime }\right)+p_{w}^{\prime }\left(p_{w1}^{\prime }f_{w1}^{\prime }+p_{w2}^{\prime }f_{w2}^{\prime }\right)\right]. \end{array}$$

These parameters in Equation (21) are calculated in several situations. The symbols of fidelity and probability are as shown in Figure 3. For cases when Charlie has successful counts and the teleported states are correct, the parameters are calculated as

(22) pc1=∑m=6,11pm∑m=6,11pmpcpc=P13+P22P13+P22pcpc=1/2,pc2=∑m=9pm∑m=9pmpcpc=P13P13pcpc=1−x221−x2241−x−x3+x441−x−x3+x4,pc3=∑m=14pm∑m=14pmpcpc=P22P22pcpc=1+x21−x21+x21−x241−x−x3+x441−x−x3+x4,

and

(23) fc1=1,fc2=I1I1B6B62=A+2−A−22=cos2θ,fc3=I1I1B9B92=A−2−A+22=cos2θ.

For cases when Charlie has successful counts, but the teleported states are wrong, the parameters are given using

(24) pw1=∑m=5,12pm∑m=5,12pmpwpw=P12+P23P12+P23pwpw=1/2,pw2=∑m=8pm∑m=8pmpwpw=P12P12pwpw=1−x21−x241+x241+x2,pw3=∑m=15pm∑m=15pmpwpw=P23P23pwpw=1+x21+x241+x241+x2,

and

(25) fw1=I1I1B3B32=I1I1B8B82=4A+A−24A+A−21+x4−2x2cosθ21+x4−2x2cosθ2=sin2θsin2θ1+x4−2x2cosθ21+x4−2x2cosθ2,fw2=I1I1B5B52=4x4A+A−24x4A+A−21+x4−2x2cosθ21+x4−2x2cosθ2=x4sin2θx4sin2θ1+x4−2x2cosθ21+x4−2x2cosθ2,fw3=I1I1B10B102=4x4A+A−24x4A+A−21+x4−2x2cosθ21+x4−2x2cosθ2=x4sin2θx4sin2θ1+x4−2x2cosθ21+x4−2x2cosθ2.

By contrast, for cases when Charlie has vacuum counts but the teleported states are correct, the parameters can be written as

(26) pc1′=∑m=10pm∑m=10pmpc′pc′=P21P21pc′pc′=1/2,pc2′=∑m=13pm∑m=13pmpc′pc′=P21P21pc′pc′=1/2.

and

(27) pc1′=1,pc2′=I1I1B9B92=A−2−A+22=cos2θ.

For cases when Charlie has vacuum counts and the teleported states are wrong, the parameters follow

(28) pw1′=∑m=4pm∑m=4pmpw′pw′=P11P11pw′pw′=1/2,pw2′=∑m=7pm∑m=7pmpw′pw′=P11P11pw′pw′=1/2.

and

(29) fw1′=I1I1B3B32=4A+A−24A+A−21+x4−2x2cosθ21+x4−2x2cosθ2=sin2θsin2θ1+x4−2x2cosθ21+x4−2x2cosθ2,fw2′=I1I1B5B52=4x4A+A−24x4A+A−21+x4−2x2cosθ21+x4−2x2cosθ2=x4sin2θx4sin2θ1+x4−2x2cosθ21+x4−2x2cosθ2.

According to the equations above, the difference of conditioned fidelity $f_{CQT}$ and non-conditioned fidelity $f_{NC}$ can be expressed as Equation (30).

(30) CP0=fCQT−fNC={1/[8(1+x2+x4)]}·{−2−1+2x−2x2+x3−3x4+x5sin2θ−4−1+2x−3x2+x3−1+x2cosθ24−1+2x−3x2+x3−1+x2cosθ21+x21+x2+2−1+2x−4x2+3x3−4x4+5x5−8x6+7x7−5x8+x9·sin2θsin2θ[1+x22(1+x4−2x2cosθ)][1+x22(1+x4−2x2cosθ)]}.

Experimentally, the superposition of coherent states of moderate value of coherent amplitudes (α∼ 2 to 3) can be generated with high fidelity in various ways, such as using Kerr-nonlinear interactions, cavity-assisted interactions, and photon subtraction from squeezed vacuum state [41]. To run a fault-tolerant quantum computation scheme using a qubit encoded in the coherent state basis $|\pm \alpha \rangle$, which is practical only when these states are nearly orthogonal, certain schemes require ${\left|\alpha \right|}^{2}>4$ [43].

**Figure 4:**
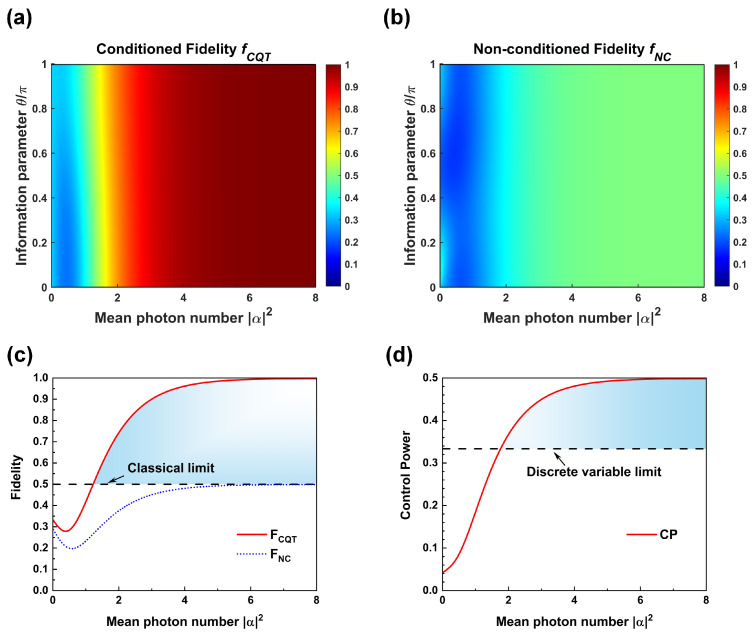
(**a**,**b**) depict the variation in the conditioned fidelity $f_{CQT}$ and non-conditioned fidelity $f_{NC}$ with respect to both mean photon number ${\left|\alpha \right|}^{2}$ and information parameter θ. (**c**) The solid red line represents conditioned fidelity $F_{CQT}$ and the dotted blue line represents non-conditioned fidelity $F_{NC}$, respectively, with respect to mean photon number ${\left|\alpha \right|}^{2}$. The colored region represents the conditioned teleportation fidelities by the protocol beyond the classical limit drawn by black dashed line. (**d**) The solid red lines represent control power CP with respect to mean photon number ${\left|\alpha \right|}^{2}$. The colored region represents the control power for the protocol beyond the reach of control power for discrete variable controlled teleportation drawn by black dashed line.

With different mean photon number ${\left|\alpha \right|}^{2}$, the variation in the conditioned fidelity $f_{CQT}$ and non-conditioned fidelity $f_{NC}$ with respect to information parameter θ are depicted in Figure 4a and Figure 4b, respectively. One can see that when the mean photon number is small (${\left|\alpha \right|}^{2}<2$), both $f_{CQT}$ and $f_{NC}$ change with information parameter θ. When ${\left|\alpha \right|}^{2}\geq 2$, the influence of information parameter θ almost disappears.

Substituting Equation (30) into Equation (17), the conditioned fidelity $F_{CQT}$ and non-conditioned fidelity $F_{NC}$ are indicated in Figure 4c. From Figure 4c, it is clear that conditioned fidelity $F_{CQT}$ and non-conditioned fidelity $F_{NC}$ both first drop and then increase with the mean photon number ${\left|\alpha \right|}^{2}$. This can be interpreted as follows. There are two factors to influence the value of fidelities $F_{CQT}$ and $F_{NC}$. One is the nonlocality of the quantum channel. The GHZ-type entangled coherent state channels will approach a highly nonlocal entangled state $\frac{1}{\sqrt{3}}\left({|1,0,0\rangle }_{2,3,4}+{|0,1,0\rangle }_{2,3,4}+{|0,0,1\rangle }_{2,3,4}\right)$ when ${\left|\alpha \right|}^{2}=0$ [44] and is approximately a vacuum state when ${\left|\alpha \right|}^{2}$ is near zero. The nonlocality of the quantum channel will drop quickly when ${\left|\alpha \right|}^{2}$ is small, which makes the fidelity have similar trend. The other factor is the mean photon number ${\left|\alpha \right|}^{2}$, which makes the value of fidelity increase when ${\left|\alpha \right|}^{2}$ further increases. $F_{CQT}$ exceeds the classical limit of teleportation 1/2 when ${\left|\alpha \right|}^{2}=1.23$, and approaches perfect protocol upper bound 1 when ${\left|\alpha \right|}^{2}\geq 5.52$. The results show that the protocol can realize an almost perfect CQT of coherent state superposition using moderate value of coherent amplitude.

The difference between $F_{CQT}$ and $F_{NC}$ depicts control power CP in Figure 4d. As shown in Figure 4d, control power increases with the mean photon number ${\left|\alpha \right|}^{2}$. When ${\left|\alpha \right|}^{2}$ is near 0, the control power is around a small positive value 0.04 instead of zero, because the quantum channels approach a highly nonlocal entangled state as mentioned above. When ${\left|\alpha \right|}^{2}=1.74$, the control power receives 1/3, which equals the CP limit of teleportation in DV controlled quantum teleportation [23]. Thus, control power in the CV controlled quantum teleportation will exceed that in DV controlled quantum teleportation. When ${\left|\alpha \right|}^{2}\to \infty$, the coherent state basis $|\pm \alpha \rangle$ are nearly orthogonal. The scheme approaches the teleportation of CV coherent state using classical channel. In this case, $F_{CQT}$ is close to 1 and $F_{NC}$ approaches the limit of fidelity in the above teleportation 1/2. Consequently, control power in Figure 4d will approaches 1−1/2=1/2 when ${\left|\alpha \right|}^{2}\geq 4.72$, which are also moderate values of coherent amplitudes.

## 4. Analysis of Robustness

In this section, the robustness analysis of continuous variable controlled quantum teleportation is discussed in aspect of the decoherence of modes due to photon absorption.

In quantum communication networks, information-carrying quantum states are transmitted across space-separated nodes via quantum channels. However, inevitable losses and noises in these channels result in decoherence of the quantum states [45]. Decoherence, which is often caused by the interaction between system and the environment, is a main factor limiting the development of the quantum information technology [45]. A more general type of decoherence is due to interactions with modes that are initially in some Gaussian state not equal to the vacuum, such as a thermal state at finite temperature. But for optical fields, the effective temperature is essentially zero, and the environment can be assumed to be in a vacuum. The decoherence due to photon absorption is considered here, which is a kind of passive loss. This loss can be modeled by a linear interaction of the mode of interest with one or more “environment” modes that are initially in the vacuum [46] as follows:

(31) $$|\alpha \rangle ⊗{|0\rangle }_{env}\to |\sqrt{\eta }\alpha \rangle ⊗{|\sqrt{1-\eta }\alpha \rangle }_{env},$$

where the second state refers to the “environment” mode or modes, and η is the survived photon proportion, which gives the proportion of photons surviving the absorption process. For simplicity, we assume that three modes are equally lost. Thus, the quantum channel becomes

(32) $$\begin{array}{c} {|GHZ,\tilde{\alpha }\rangle }_{2,3,4}={\left[2\left(1-{\tilde{x}}^{6}\right)\right]}^{-1/2}\cdot \left({|\tilde{\alpha },\tilde{\alpha },\tilde{\alpha }\rangle }_{2,3,4}-{|-\tilde{\alpha },-\tilde{\alpha },-\tilde{\alpha }\rangle }_{2,3,4}\right), \end{array}$$

where $\tilde{\alpha }=\sqrt{\eta }\alpha$, $\tilde{x}=e^{-{\left|\tilde{\alpha }\right|}^{2}}$. In the presence of decoherence, the information states are of the form

(33) $${|\tilde{I}\rangle }_{1}=A_{+}{|\tilde{E}\rangle }_{1}+A_{-}{|\tilde{O}\rangle }_{1},$$

where

(34) $$\begin{array}{c} |\tilde{E}\rangle ={\left[\sqrt{2\left(1+{\tilde{x}}^{2}\right)}\right]}^{-1}\left(|\tilde{\alpha }\rangle +|-\tilde{\alpha }\rangle \right),\\ |\tilde{O}\rangle ={\left[\sqrt{2\left(1-{\tilde{x}}^{2}\right)}\right]}^{-1}\left(|\tilde{\alpha }\rangle -|-\tilde{\alpha }\rangle \right). \end{array}$$

**Figure 5:**
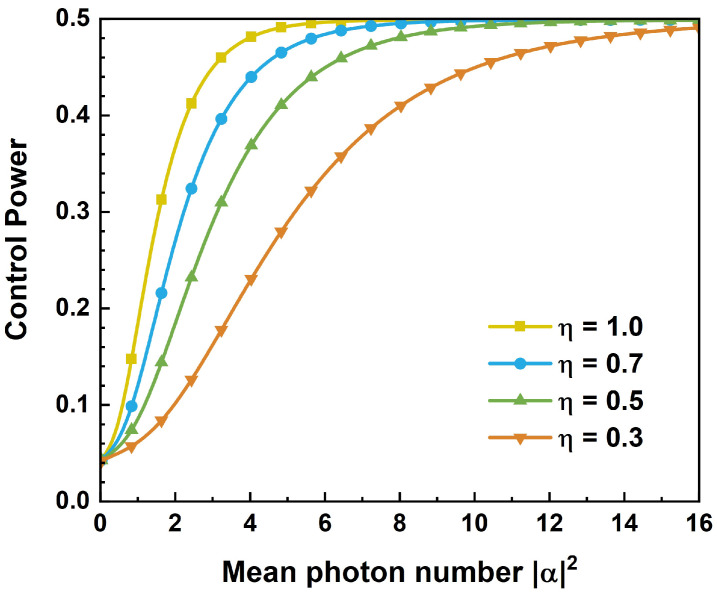
Control power for different values of the survived photon proportion η as a function of mean photon number ${\left|\alpha \right|}^{2}$.

The following steps are the same as Section 2 and Section 3. The results of control power for different values of the survived photon proportion η are plotted in Figure 5. One can see from Figure 5 that the increasing photon loss will reduce the improving ability of the control power. With a large ${\left|\alpha \right|}^{2}$, the control power is approaching the upper bound of 1/2. This upper bound is independent of the decoherence due to photon absorption.

## 5. Summary and Outlook

In summary, we define a measure to quantify the control power in continuous variable controlled quantum teleportation via GHZ-type entangled coherent state channels. Compared with the experimental protocol proposed by Furusawa et al. [27], the protocol in this paper makes a difference in the classification of result of photon counting and in the planning for unitary transformations to be performed by Bob [47]. Then, this protocol can achieve nearly perfect teleportation fidelity when the control power approach upper bound. Our results show that control power in the CV controlled quantum teleportation increases with the mean photon number ${\left|\alpha \right|}^{2}$ and then approaches an upper bound 1/2. The upper bound of control power will exceed that in DV controlled quantum teleportation (1/3) [23].

We also analyze the robustness with photon absorption. One can see that the upper bound of the control power has robustness with different survived photon proportions. When the photon loss increases, the improving ability of the control power will descend and the control power reaches the upper bound at a larger mean photon number. The results illuminate the role of control power in multipartite continuous variable quantum information processing and provide a criterion for evaluating the quality of quantum communication networks.

In regard to the channel loss, there are two distinct types of quantum channels: the lossy channel and the noisy channel. In a lossy but noiseless (without excess noise) quantum channel, the noise arising from loss is solely the vacuum noise, which corresponds to a zero-temperature environment [9]. In a noisy channel, the excess noise higher than the vacuum noise exists [9]. Disentanglement of light field when the excess noise exists in the quantum channel on tripartite entangled state is demonstrated [45]. The effects of fiber channel loss are discussed in CV quantum key distribution [39,40]. We hope the influence for the control power in CV CQT protocol in the lossy channel and the noisy channel can be explored in the future works.

## Data Availability

Data is contained within the article.

## Appendix A. Diverse Possible Results of PC Measurement

In this appendix, the PC measurement amounting to a total of 15 mutually exclusive outcomes, which we shall abbreviate by cases m=1,2,...,15 in Table A1. The unitary operations can be represented as

(A1) $$\begin{array}{c} U_{1}={|E\rangle }_{4}\langle O|+{|O\rangle }_{4}\langle E|,\\ U_{2}={|E\rangle }_{4}\langle O|-{|O\rangle }_{4}\langle E|,\\ U_{3}={|E\rangle }_{4}\langle E|-{|O\rangle }_{4}\langle O|, \end{array}$$

and *I* refers to identity transformation.

After utilizing the corresponding unitary operations by Bob, the teleported states can be obtained by

(A2) $$\begin{array}{c} |T_{1}\rangle ={|O\rangle }_{4},\\ |T_{2}\rangle =N_{4}\left[A_{+}\left(1-x^{2}\right){|E\rangle }_{4}+A_{-}\left(1+x^{2}\right){|O\rangle }_{4}\right],\\ |T_{3}\rangle =A_{+}{|E\rangle }_{4}+A_{-}{|O\rangle }_{4}, \end{array}$$

where $N_{4}={\left(1+x^{4}-2x^{2}\cos\theta \right)}^{-1/2}$ denotes normalization parameter of mode 4 with Bob.

**Table A1:** Diverse possible results of PC measurement in modes 6 and 8 with Alice, and mode 3 with supervisor Charlie. For each case, the corresponding probability of occurrence, required unitary operation, teleported state and fidelity are given.

*m*	Alice’s PC Results		Charlie’s PC Result	Probability	Unitary Operation	Bob’s State	Teleported State	Fidelity
	(6)	(8)	(3)			(4)		
1	0	0	0	$P_{01}$	$U_{1}$	$|B_{1}\rangle$	$|T_{1}\rangle$	$F_{0}$
2	0	0	NZE	$P_{02}$	$U_{1}$	$|B_{1}\rangle$	$|T_{1}\rangle$	$F_{0}$
3	0	0	ODD	$P_{03}$	*I*	$|B_{2}\rangle$	$|T_{1}\rangle$	$F_{0}$
4	NZE	0	0	$P_{11}$	$U_{1}$	$|B_{3}\rangle$	$|T_{2}\rangle$	$F_{1}$
5	NZE	0	NZE	$P_{12}$	$U_{1}$	$|B_{3}\rangle$	$|T_{2}\rangle$	$F_{1}$
6	NZE	0	ODD	$P_{13}$	*I*	$|B_{4}\rangle$	$|T_{3}\rangle$	1
7	0	NZE	0	$P_{11}$	$U_{2}$	$|B_{5}\rangle$	$|T_{2}\rangle$	$F_{1}$
8	0	NZE	NZE	$P_{12}$	$U_{2}$	$|B_{5}\rangle$	$|T_{2}\rangle$	$F_{1}$
9	0	NZE	ODD	$P_{13}$	$U_{3}$	$|B_{6}\rangle$	$|T_{3}\rangle$	1
10	ODD	0	0	$P_{21}$	*I*	$|B_{7}\rangle$	$|T_{3}\rangle$	1
11	ODD	0	NZE	$P_{22}$	*I*	$|B_{7}\rangle$	$|T_{3}\rangle$	1
12	ODD	0	ODD	$P_{23}$	$U_{1}$	$|B_{8}\rangle$	$|T_{2}\rangle$	$F_{1}$
13	0	ODD	0	$P_{21}$	$-U_{3}$	$|B_{9}\rangle$	$|T_{3}\rangle$	1
14	0	ODD	NZE	$P_{22}$	$-U_{3}$	$|B_{9}\rangle$	$|T_{3}\rangle$	1
15	0	ODD	ODD	$P_{23}$	$-U_{2}$	$|B_{10}\rangle$	$|T_{2}\rangle$	$F_{1}$
